# Colorectal Polyp Prevalence According to Alcohol Consumption, Smoking and Obesity

**DOI:** 10.3390/ijerph17072387

**Published:** 2020-03-31

**Authors:** Kyujin Lee, Yong Hwan Kim

**Affiliations:** 1 Institute of Sports Science, Seoul National University, Seoul 08826, Korea; my993286@snu.ac.kr; 2 Department of Physical Education, Gangneung-Wonju National University, Gangneung-si 25457, Korea

**Keywords:** colorectal polyp, alcohol, smoking, obesity, odds ratio

## Abstract

This study aimed to analyze colorectal polyp prevalence associated with health behavior. Data from 1180 Korean men (young adult (YA), aged 40–49; middle age (MA) aged 50–59; old aged (OA), aged 60–79 years) were collected. Health behavior included alcohol consumption, smoking status, and obesity. Obesity was determined using body mass index (BMI) and waist circumference (WC). Odds ratio (OR) was calculated by logistic regression. The prevalence of polyps increased for current smokers by 2.642 times in the YA group, 3.468 times in the MA group, and 3.104 times in the OA group compared to the never-smokers. The OR for WC increased in subjects with obesity by 1.514 in the MA and 1.451 in the OA group compared to normal. The prevalence of three or more polyps increased with WC obesity by 2.3 times in YA, 2.2 times in MA, and 1.9 times in OA compared to normal WC. Therefore, smoking cessation and obesity management may reduce the risk of colorectal polyps.

## 1. Introduction

Cancer was the leading cause of death in Korea in 2018, and colorectal cancer has the third highest mortality rate among cancers [1]. The incidence rate per 100,000 individuals increased from 21.2 in 1999 to 31.9 in 2016 [2]. This high incidence of colorectal cancer has made it the third most common cancer in the world as well as in Korea [3].

Colorectal polyps are lesions that protrude over the intestinal mucosa, and most polyps are adenomas, which are known to be precursors of colorectal cancers [4]. Since the prevalence of colorectal polyps in the Korean population is relatively common (26.7% of those in their 40 s and 37.8% of those in their 50 s), it is necessary to detect and treat early symptoms and prevent high-risk factors for cancer [5].

However, the risk factors for colorectal polyps are unclear. Recent studies revealed the male sex and increasing age are risk factors [6]. Additionally, modifiable factors, such as obesity, high alcohol consumption, and smoking, are known to increase the risk of developing colorectal polyps. Accordingly, quitting smoking, controlling alcohol consumption, and losing weight appeared to be effective prevention strategies for colorectal polyp prevention [7,8,9,10,11].

A study involving middle-aged individuals reported that smokers who consumed 20 packs per year had a 1.87 times higher polyp prevalence than did nonsmokers [12]. Moreover, individuals who consumed alcohol >3 days a week showed a 2.01-fold increase in adenomatous polyp prevalence compared to those who did not consume alcohol [13]. Furthermore, obesity-related factors such as BMI, total fat area, subcutaneous fat area, and visceral fat area are reported to have a positive correlation with polyp number [14].

However, most previous studies have either specific age groups or an extremely wide range of ages. Therefore, although this is a cross-sectional study, subjects were divided into young adult (YA), middle age (MA), and old age (OA) groups. We also analyzed the differences in colorectal polyp prevalence based on health behaviors, namely obesity, smoking status, and alcohol consumption.

## 2. Materials and Methods

### 2.1. Subjects

This study examined Korean men aged 40–79 years who visited health screening centers in Seoul, Korea. A health screening service and colonoscopy were performed as per the voluntary participation. Overall, 1543 subjects were included from January to December 2017, but only 1180 subjects’ data were collected in the final analysis. Individuals who did not agree to participate in the study and those whose examination and questionnaire were incomplete were excluded. The participants were divided into three groups according to age: YA group (n = 360, 40–49 years), MA group (n = 484, 50–59 years), and OA group (n = 264, 60–79 years). Only the medical results of individuals who agreed to use the test results for research purposes were included in the study for ethical considerations. To protect personal information, researchers used only data related to the research and deleted personally identifiable information.

### 2.2. Health Behavior Questionnaire: Alcohol Consumption and Smoking Status

The questionnaires included questions on medical history, alcohol consumption, and smoking status. Regarding alcohol consumption, the frequency of drinking per week and alcohol volume were determined. The risk level was classified according to the guidelines of the World Health Organization (WHO) [15]. Depending on the type of alcohol, the pure alcohol amount was calculated: 1–40 g/day was classified as low risk, 41–60 g/day, as moderate risk, and ≥61 g/day, as high risk [15]. Regarding smoking status, individuals who never smoked were classified as “Never,” individuals who quit, as “Former,” and individuals who currently smoked, as “Current.” The daily smoking amount was measured in terms of the number of packs; therefore, it was categorized as 0.5 pack, 1 pack, 1.5 pack, and ≥2 packs.

### 2.3. Obesity

Obesity was diagnosed on the basis of BMI and waist circumference (WC). Based on the WHO guidelines, BMI of ≥24.9 was considered normal, 25.0–29.9 was overweight, and ≥30.0 was obese [16]. According to the Korean Society for the Study of Obesity, a WC of ≥90.0 cm indicated obesity [17].

### 2.4. Colonoscopy

A colonoscopy was performed by a gastroenterologist with the participants in a sleep-induced condition in the morning; the participants had fasted since dinner on the previous day. The participants received polyethylene glycol electrolyte solution and water for colon cleansing. The location, size, and number of all polyps were determined during colonoscopy. All polyps were completely removed and then pathologically examined. The polyps were adenomatous and hyperplastic. The first analysis examined the prevalence of polyps. The second analysis was only for people with polyps, and the presence of ≥3 polyps was defined as a high risk [18]. Each analysis calculated the prevalence of polyps according to health behavior.

### 2.5. Statistics Analysis

SPSS 25.0 software (SPSS IBM, New York, NY, USA) was used for the data analysis. Continuous variables, such as height, weight, and BMI, were expressed as mean and standard deviation. To confirm the difference according to the presence of polyps, an independent t-test was conducted in each age group. Moreover, a chi-square test was used to analyze categorical variables, such as alcohol consumption and smoking status. To determine the odds ratio (OR), the prevalence was analyzed by logistic regression. Multiple collinearity was evaluated by performing multiple regression analysis with stepwise to calculate the adjusted variables and odds ratio. The adjustment variables included WC, alcohol amount, and smoking status. A *p*-value of <0.05 indicated statistical significance.

## 3. Results

### 3.1. Characteristics of Subjects

Table 1 shows the result of the analysis of the general characteristics of colorectal polyps. The incidence of colorectal polyps was 42.5% in the YA group, 55.0% in the MA group, and 66.3% in the OA group. In the MA group, those with polyps had higher WC (*p* < 0.05) than those without polyps. In the OA group, BMI was significantly higher in those with polyps than in those without.

Table 2 shows the results of the analysis of the significant difference as per the chi-square test by clustering the variables. There was a significant difference in smoking status between all ages, and there was a significant difference in smoking amount in YA and MA. WC showed significant results in the MA group, and BMI in the OA group. Alcohol amount showed significant difference according to the presence or absence of polyps in the YA and MA group (*p* < 0.05).

### 3.2. Prevalence of >1 Polyp Related to Obesity, Smoking and Alcohol Consumption

Table 3 shows the result of the analysis of polyp prevalence based on obesity, smoking, and alcohol consumption. Based on the WC, obese participants showed 1.515 times and 1.451 times higher polyp prevalence in the MA and OA groups, respectively, than in the normal group. However, there was no significant result in WC obesity and polyps in the YA group. Current smokers had a higher prevalence of polyps by 2.642 times in the YA group, 3.468 times in the MA group, and 3.104 times in the OA group than in the never smoked group. Even in those who had quit smoking, MA and OA increased the prevalence of polyps by 2.4 and 1.8 times compared to never-smokers. Alcohol showed significant results only in the MA group, and the high-risk group had a 1.7 times higher polyp prevalence than the low-risk group.

### 3.3. Prevalence of ≥3 Polyps Related to Obesity, Smoking and Alcohol Consumption

Table 4 shows the results for individuals with polyps. Persons with three or more polyps were classified as high risk and analyzed for prevalence of polyps according to health behavior. Obesity based on WC significantly increased the prevalence of high-risk polyps at all ages. The OR values were 2.379 times in the YA group, 2.021 times in the MA group, and 1.978 times in the OA group than the normal group. In the YA, high-risk drinkers had a 3.540 times higher risk of polyps than low-risk drinkers. In the case of high-risk polyp prevalence, there was no significant increase in the prevalence according to smoking.

## 4. Discussion

Colorectal cancer is one of the most common diseases worldwide with the third highest incidence rate and an extremely high mortality rate [3]. Some nonmodifiable factors that cause colorectal cancer include sex, aging, and heredity. However, some modifiable lifestyle factors, such as exercise and physical activity, quitting smoking, and obesity management can also positively affect etiology [19,20]. Furthermore, any healthcare services to screen, remove, and prevent polyps are important to prevent colorectal cancer as a polyps are precursors to colorectal cancer [4]. This study showed that the incidence rates of polyps were 42.5% in the YA group, 55.0% in the MA group, and 66.3% in the OA group. This indicates that colorectal polyps are influenced by an increase in age [21].

One of the major findings of this study is that there is a significant association between smoking and colorectal polyp prevalence. This is in line with previous studies that have shown a correlation between smoking and polyp prevalence. The colorectal polyp prevalence increased by 3.40 times in current smokers compared to “never” smokers. These results are similar to those of previous studies. According to a study by Botteri et al., people who quit smoking had a 1.47-fold increase in polyp prevalence compared to “never” smokers [22]. Furthermore, a study on the American population also showed that polyp prevalence OR increased from 1.1 to 2.0 times with daily consumption of <0.5 packs of cigarettes [23]. In this study, the prevalence of polyps was higher in former smokers of MA and OA. These results were similar to previous studies. A study by Shrubsole et al., reported that former smokers’ prevalence of polyps increased by 2.8 times and current smokers increased by 7.7 times compared to never-smokers [24]. In another study, the former smokers’ prevalence of polyps increased by 1.18–1.42 times compared to non-smoker [25]. Tobacco contains Group 1 carcinogens, such as benzene, in addition to nicotine and tar [26]. However, a detailed study of biomedical mechanisms will be needed to identify the specific substances that cause polyps.

In this study, colorectal polyps did not show a significant association with alcohol frequency (Table 2). Based on daily consumption, it did not show any significance in the YA and OA groups. In alcohol-related analyses, only the alcohol amount of MA was significant, and the OR value increased to 1.7 times in the high-risk group as compared to the low-risk (Table 3). These results were similar to those in previous studies. A study on Taiwanese individuals in Asia showed that polyp prevalence increased by 2.01 times in those who consumed alcohol than in those who did not consume alcohol [13]. A study by Gardou et al. showed that excessive alcohol consumption increases the prevalence of developing high-risk polyps of >10 mm in size [27]. However, a study on the Caucasian population showed that low alcohol consumption of >7 g/day did not significantly affect polyp incidence [28].

Although previous studies have not reported consistent results for the function of alcohol, the International Agency for Research on Cancer under the WHO has designated alcohol as a primary carcinogen. Alcohol consumption is known to cause cancers, such as breast cancer mainly in females, and colorectal, laryngeal, liver, esophageal, oral, and pharyngeal cancers [29].

WC, used in this study to examine obesity, was found to be related to the presence of polyps in the MA (1.514 times) and OA groups (1.451 times). For high-risk factors with ≥3 polyps, all age groups showed significant results only in WC variables. This obesity-related factor indicates that not only maintaining a normal BMI, but also keeping WC at ≤89.9 cm is important. Previous studies found that WC ≥90.0 cm increases the risk of polyp incidence by 1.59 times [30].

Colorectal cancer development is sustained by different factors including the diet and gut microbiota [31]. Gut microbiota play an immune function against pathogenic bacteria colonization and invasion, and contributes to the maintenance of the intestinal epithelium integrity [32]. Microbiota variations could be caused by host-related factors including BMI, exercise frequency, and dietary habits. Concerning the last aspect, recent studies have shown that diet has an important role in shaping the gut microbiota [33,34,35]. According to previous studies that reported the correlation between colorectal and dietary factors [36], we believe dietary factors influenced our results. However, our study did not discuss the relation between the dietary and colorectal polyps because dietary variables were not directly measured.

This study has the following limitations: First, because this study was a cross-sectional study, there was a limit to explaining the causal relationship between health behavior and colorectal polyp prevalence. Second, this study did not investigate the smoking period and quitting period. One study showed that 55.3% of male smokers attempt to quit smoking, but only 7.2% maintained smoking cessation [37]. Smoking and smoking cessation periods were not investigated in this study, so their effects were not reflected in the results analysis. Third, since this study did not reflect the socioeconomic status in the result, it cannot represent the general social classes. Generally, individuals with better economic conditions tend to participate in obesity management and exercise, and this leads to a low rate of smoking and alcohol consumption [38]. People at economically high levels of society are likely to find and remove polyps with increased access to expensive colonoscopy [39]. Finally, colorectal polyps are affected by nutrition status [40]. However, in this study, since diet was not investigated, its effects were not reflected in the analysis. Future studies should be conducted to complement the limitations and also to include women.

## 5. Conclusions

The incidence of colorectal polyps increased with age. Current and past smokers also have increased polyp prevalence. In addition, high waist circumference is a factor in increasing the prevalence of colorectal polyps. Therefore, maintaining low waist circumference and nonsmoking status could help to prevent colorectal polyps.

## Figures and Tables

**Table 1:** Characteristics of subjects.

	YA (n = 360)		MA (n = 484)		OA (n = 264)	
Variables	Non-polyp 207 (57.5%)	Polyp 153 (42.5%)	Non-polyp 218 (45.0%)	Polyp 266 (55.0%)	Non-polyp 89 (33.7%)	Polyp 175 (66.3%)
Age, years	44.9 ± 2.8	45.8 ± 2.6 *	53.8 ± 2.8	54.4 ± 2.9 *	65.7 ± 3.9	65.7 ± 4.0
Height, cm	170.7 ± 5.3	170.7 ± 5.1	169 ± 5.6	169.5 ± 5.6	168.6 ± 5.5	167.8 ± 5.9
Weight, kg	72.7 ± 9.1	74.3 ± 9.2	72.4 ± 9	74.0 ± 9.5	69.4 ± 9.3	71.2 ± 8.4
BMI, kg/m^2^	24.9 ± 2.7	25.5 ± 2.7	25.3 ± 2.6	25.7 ± 2.8	24.4 ± 3.0	25.3 ± 2.4 *
WC, cm	84.4 ± 6.9	88.0 ± 5.8	85.6 ± 6.9	89.3 ± 7.1 *	85.6 ± 8.2	87.4 ± 6.6

* *p* < 0.05; express to average ± standard deviation; BMI, body mass index; WC, waist circumference; WHtR, waist to height ratio; YA, young adult; MA, middle age; OA, old age.

**Table 2:** Difference of variables classification.

		YA (n = 360)		MA (n = 484)		OA (n = 264)	
Variables	classification	Non-polyp 207 (57.5%)	Polyp 153 (42.5%)	Non-polyp 218 (45.0%)	Polyp 266 (55.0%)	Non-polyp 89 (33.7%)	Polyp 175 (66.3%)
BMI, kg/m^2^			*p* = 0.059		*p* = 0.338		*p* = 0.017*
	Normal	49 (23.5%)	26 (16.8%)	38 (17.5%)	34 (12.8%)	28 (31.4%)	28 (16.0%)
	Overweight	61 (29.4%)	36 (23.5%)	59 (26.9%)	70 (26.5%)	18 (19.8%)	42 (24.3%)
	Obesity	97 (47.1%)	91 (59.7%)	121 (55.7%)	161 (60.7%)	43 (48.8%)	105 (59.8%)
WC, cm			*p* = 0.098		*p* = 0.028 *		*p* = 0.083
	Normal	157 (75.8%)	104 (68%)	159 (72.9%)	169 (63.5%)	64 (71.9%)	107 (61.1%)
	Obesity	50 (24.2%)	49 (32%)	59 (27.1%)	97 (36.5%)	25 (28.1%)	68 (38.9%)
Alcohol frequency			*p* = 0.086		*p* = 0.207		*p* = 0.705
	0–1/week	63 (30.4%)	57 (37.3%)	104 (47.7%)	105 (39.5%)	49 (55.1%)	92 (52.6%)
	2–3/week	82 (39.6%)	42 (27.5%)	66 (30.3%)	84 (31.6%)	24 (27.0%)	41 (23.4%)
	5–6/week	40 (19.3%)	39 (25.5%)	32 (14.7%)	47 (17.7%)	9 (10.1%)	22 (12.6%)
	7/week	22 (10.6%)	15 (9.8%)	16 (7.3%)	30 (11.3%)	7 (7.9%)	20 (11.4%)
Alcohol amount per day, risk			*p* = 0.404		*p* = 0.040 *		*p* = 0.793
	Low	108 (51.9%)	67 (43.7%)	152 (69.7%)	152 (57.0%)	69 (77.3%)	135 (77.2%)
	Medium	39 (18.8%)	31 (20.4%)	26 (11.8%)	45 (17.0%)	6 (6.8%)	8 (4.3%)
	High	60 (29.2%)	55 (35.9%)	40 (18.5%)	69 (26.1%)	14 (15.9%)	32 (18.5%)
Smoking status			*p* = 0.026 *		*p* < 0.001 *		*p* = 0.018 *
	Never	38 (18.4%)	16 (10.5%)	43 (19.7%)	23 (8.6%)	27 (30.3%)	39 (22.3%)
	Quitting	82 (39.6%)	53 (34.6%)	109 (50%)	132 (49.6%)	49 (55.1%)	83 (47.4%)
	Current	87 (42%)	84 (54.9%)	66 (30.3%)	111 (41.7%)	13 (14.6%)	53 (30.3%)
Smoking amount per day, pack			*p* = 0.047 *		*p* = 0.007 *		*p* = 0.316
	0	38 (18.5%)	16 (10.7%)	44 (20.3%)	24 (8.8%)	28 (31.8%)	40 (22.7%)
	0.5	37 (18.0%)	18 (12.0%)	41 (18.9%)	48 (18.1%)	15 (16.5%)	26 (15.1%)
	1.0	68 (32.7%)	62 (41.3%)	74 (34.0%)	101 (38.1%)	28 (31.8%)	58 (33.1%)
	1.5	50 (24.4%)	38 (25.3%)	36 (16.5%)	56 (21.2%)	14 (15.3%)	32 (18.0%)
	≥2	13 (6.3%)	16 (10.7%)	23 (10.4%)	37 (13.8%)	4 (4.7%)	19 (11.0%)

* *p* < 0.05; express to number (%); BMI, body mass index; WC, waist circumference; WHtR, waist to height ratio; YA, young adult; MA, middle age; OA, old age. BMI normal ≤24.9, overweight 25.0–29.9, obesity ≥30.0. Waist circumference normal ≤89.9, obesity ≥90.0. Waist to height ratio normal ≤0.49, obesity ≥0.50. Pure alcohol consumption risk low 1–40 g/day, medium 41–60 g/day, ≥61 g/day.

**Table 3:** Colorectal polyp and health behavior (polyp number 0 vs. ≥1).

		YA (n = 360)	MA (n = 484)	OA (n = 264)
Variables	Group	OR (CI 95%)	OR (CI 95%)	OR (CI 95%)
WC, cm	Normal	Reference	Reference	Reference
	Obesity	1.517 (0.947–2.432)	1.514 (1.023–2.240) *	1.451 (1.220–1.700) *
Alcohol amount per day, risk	Low	Reference	Reference	Reference
	Medium	1.391 (0.701–2.757)	1.784 (0.878–3.625)	0.638 (0.135–3.014)
	High	1.607 (0.894–2.887)	1.772 (1.076–3.215) *	1.163 (0.441–3.069)
Smoking status	Never	Reference	Reference	Reference
	Former	1.558 (0.783–3.100)	2.464 (1.387–4.377) *	1.829 (1.273–2.628) *
	Current	2.642 (1.348–5.181) *	3.468 (1.902–6.324) *	3.104 (2.104–4.579) *

* *p* < 0.05; OR, odds ratio; CI, confidence interval; BMI, body mass index; WC, waist circumference; YA, young adult; MA, middle age; OA, old age. Waist circumference normal ≤89.9, obesity ≥90.0. Pure alcohol consumption risk low 1–40 g/day, medium 41–60 g/day, ≥61 g/day according to WHO [15]. The adjustment variables included WC, alcohol amount and smoking status.

**Table 4:** Colorectal polyp and health behavior (polyp number 1–2 vs. ≥3).

		YA (n = 153)	MA (n = 266)	OA (n = 175)
Variables	Group	OR (CI 95%)	OR (CI 95%)	OR (CI 95%)
WC, cm	Normal	Reference	Reference	Reference
	Obesity	2.379 (1.117–5.069) *	2.021 (1.167–3.500) *	1.978 (1.242–3.149) *
Alcohol amount per day, risk	Low	Reference	Reference	Reference
	Medium	2.648 (0.748–9.378)	0.613 (0.202–1.862)	1.240 (0.244–7.864)
	High	3.540 (1.226–7.227) *	1.923 (0.952–3.886)	1.288 (0.437–3.799)
Smoking status	Never	Reference	Reference	Reference
	Former	0.792 (0.254–2.468)	2.153 (0.727–6.377)	1.098 (0.580–2.080)
	Current	1.304 (0.448–3.794)	2.839 (0.950–8.481)	1.102 (0.565–2.148)

* *p* < 0.05; OR, odds ratio; CI, confidence interval; WC, waist circumference; YA, young adult; MA, middle age; OA, old age. Waist circumference normal ≤89.9, obesity ≥90.0. Pure alcohol consumption risk low 1–40 g/day, medium 41–60 g/day, ≥61 g/day according to WHO [15]. The adjustment variables included WC, alcohol amount and smoking status.
